# Small is beautiful: climate-change science as if people mattered

**DOI:** 10.1093/pnasnexus/pgac009

**Published:** 2022-03-02

**Authors:** Regina R Rodrigues, Theodore G Shepherd

**Affiliations:** Department of Oceanography, Federal University of Santa Catarina, Florianópolis, SC 88040-900, Brazil; Department of Meteorology, University of Reading, Reading RG6 6BB, UK

## Abstract

There is a widely accepted gap between the production and use of climate information. It is also widely accepted that at least part of the reason for this situation lies in the challenge of bridging between what may be characterized as ‘‘top-down’’ approaches to climate information on the global scale, and local decision contexts, which necessarily take a ‘‘bottom-up’’ perspective, in which climate change is just one factor among many to consider. We here reflect on the insights provided in a different context—that of economics—by E.F. Schumacher in his celebrated book *Small is Beautiful* (1973), to see what light they might shed on this challenge, with a focus on climate-change science for adaptation. Schumacher asked how economics might look if it was structured “as if people mattered”. We ask the same question of climate-change science, and find many parallels. One is the need to grapple with the complexity of local situations, which can be addressed by expressing climate knowledge in a conditional form. A second is the importance of simplicity when dealing with deep uncertainty, which can be addressed through the use of physical climate storylines. A third is the need to empower local communities to make sense of their own situation, which can be addressed by developing ‘‘intermediate technologies’’ that build trust and transparency. Much of climate-change science is necessarily big science. We argue that in order to make climate information useable for adaptation, it is also necessary to discover the beauty of smallness.

## Introduction

As climate change increasingly permeates public discourse, the relevance of climate-change science continues to grow across many different sectors of society. There has long been a call for useable (or actionable) climate information, beginning already 50 years ago (1), and formalized in the launch of the World Meteorological Organization's Global Framework for Climate Services a decade ago (2). Yet despite this awareness and global effort, it is widely accepted that there is a significant gap between the production and use of climate information (3, 4). In the case of climate services—defined by NRC (5) as “The timely production and delivery of useful climate data, information, and knowledge to decision makers”—Findlater et al. (6) argue that the gap results in part from the focus on better data rather than on better decision-making. Even if user-informed, such a ‘‘top-down’’ approach adopts disciplinary-based measures of scientific quality, and is inevitably driven by the climate scientists themselves. It thus violates the core principles of co-production, which has a rich legacy in sustainability studies (7). Findlater et al.’s critique aligns with Coen's (1) conclusion that in order to be useable, climate-change science has to break with the traditional research/assessment/policy paradigm, and “[bring] into existence a community of users”.

**Fig. 1.**
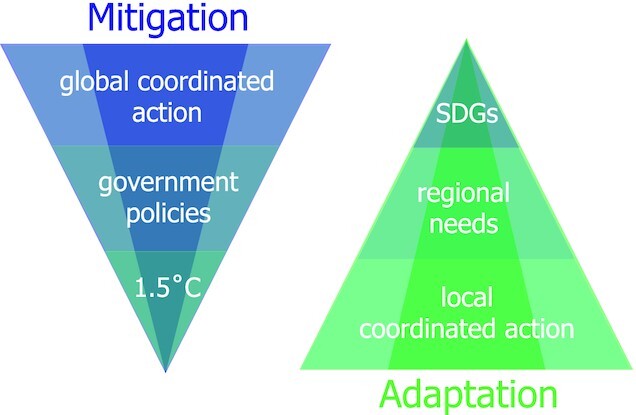
Contrast between the ‘‘top-down’’ approach in climate-change science, which is needed for mitigation action, and the ‘‘bottom-up’’ approach needed for adaptation action.

Whilst a ‘‘top-down’’ approach is necessary for a global coordinated action of government policies to stay under the Paris agreement target of 1.5°C (mitigation), the local nature of adaptation action requires the sort of ‘‘bottom-up’’ approach that Coen (1) describes, while the global target is more aspirational, represented broadly by the Sustainable Development Goals (SDGs; Fig. 1), which concern vulnerability. (The Intergovernmental Panel on Climate Change [IPCC] defines “Adaptation” as “the process of the adjustment in natural or human systems in response to actual or expected climatic stimuli or their effects, which moderates harm or exploits beneficial opportunities,” and “Mitigation” as “human intervention to reduce the sources or enhance the sinks of greenhouse gases” (8). Climate information for adaptation aims to reduce climate vulnerability.) To adopt such a ‘‘bottom-up’’ approach is, however, a radical proposition from a traditional climate science perspective. For example, Guldi (9) argues for democratizing the collection and production of climate information, which goes against the oft-heard mantra of producing ‘‘authoritative” climate information (e.g. (10)). In the broader context of sustainability, Wuelser et al. (11) argue for the need to find ways of building common ground and constructing useable knowledge from single case studies, whilst Szabo et al. (12) point out the need for spatially disaggregated data in translating the SDGs into practical actions to reduce vulnerability in tropical delta regions. Both requirements go against the natural tendency of climate scientists to aggregate data in the search for general explanations (13), and necessarily embed climate-change science within a social context. Ultimately, the bottom-up imperative argues for looking at climate-change science from a *human* perspective. Coen (1) captures this spirit when she suggests that in order to be useable, climate-change science needs to “[institutionalize] research as care…. care for data and its analysis, and care for people and their relationships.”

Nearly 50 years ago, the economist E.F. Schumacher published his celebrated book *Small is Beautiful* (1973), where he asked how economics might look if it was structured “as if people mattered.” His question was motivated by the mismatch between the values enshrined in accepted economic principles and what people actually care about. The emphasis on *care* is echoed by the quotation from Coen (1) in the previous paragraph. There might seem to be little connection between the practice of climate-change science and that of economics. (In this piece we use the term “climate-change science” to refer to the physical basis of climate change, as reflected in the composition of Working Group I of the IPCC, which might be viewed as the basic science behind climate change. This usage is for succinctness and is not intended to be normative or exclusionary. In particular, it is not meant to suggest that applied science (such as engineering or health) is not part of climate-change science in its wider sense.) However, climate-change science is anchored in physics, and the philosopher of science Nancy Cartwright (14) singled out physics and economics as the two ‘‘imperialist’’ sciences (one natural, the other social) for their tendency to seek general explanations of phenomena within their domain. (Mirowski (15) discusses the historical interactions between the two disciplines.) This parallel suggests that it may be worthwhile to ask Schumacher's question of climate-change science. (By doing so we are not suggesting that economics holds any privileged role in addressing climate risk, nor are we making any judgement on the practice of economics.)

Thus, in this article, we ask how climate-change science might look if it was structured “as if people mattered.” This is not a new question to be asking, as it was the question asked by postnormal science (16). More recently, Hulme (17) examined its implications for what are perceived to be the knowledge gaps, and Schipper et al. (18) examined its implications for thinking about interdisciplinarity. Here, we tackle the question by considering a number of concepts from *Small is Beautiful*, and translating them into the context of climate-change science, which we believe provides yet another angle on the issue. We do not address the practice of use of climate information for decision-making, which raises political, social, and economic issues (19)—and is beyond our expertise—but confine ourselves to how the science itself can be reconfigured to be more fit for purpose. Nor do we argue, as Sobel (20) has, that climate science should be prioritized to address adaptation. Instead, we argue for better ways of constructing climate information for adaptation. Our target audience is climate scientists who are interested in making their research useable in the context of climate adaptation and local climate risk. To make things concrete, we begin with a case study which illustrates the nature of the challenge. We then consider three distinct dimensions of the challenge: grappling with complexity, the importance of simplicity, and empowering local communities. The paper concludes with a synthesis.

## Case study: eastern South America

**Fig. 2.**
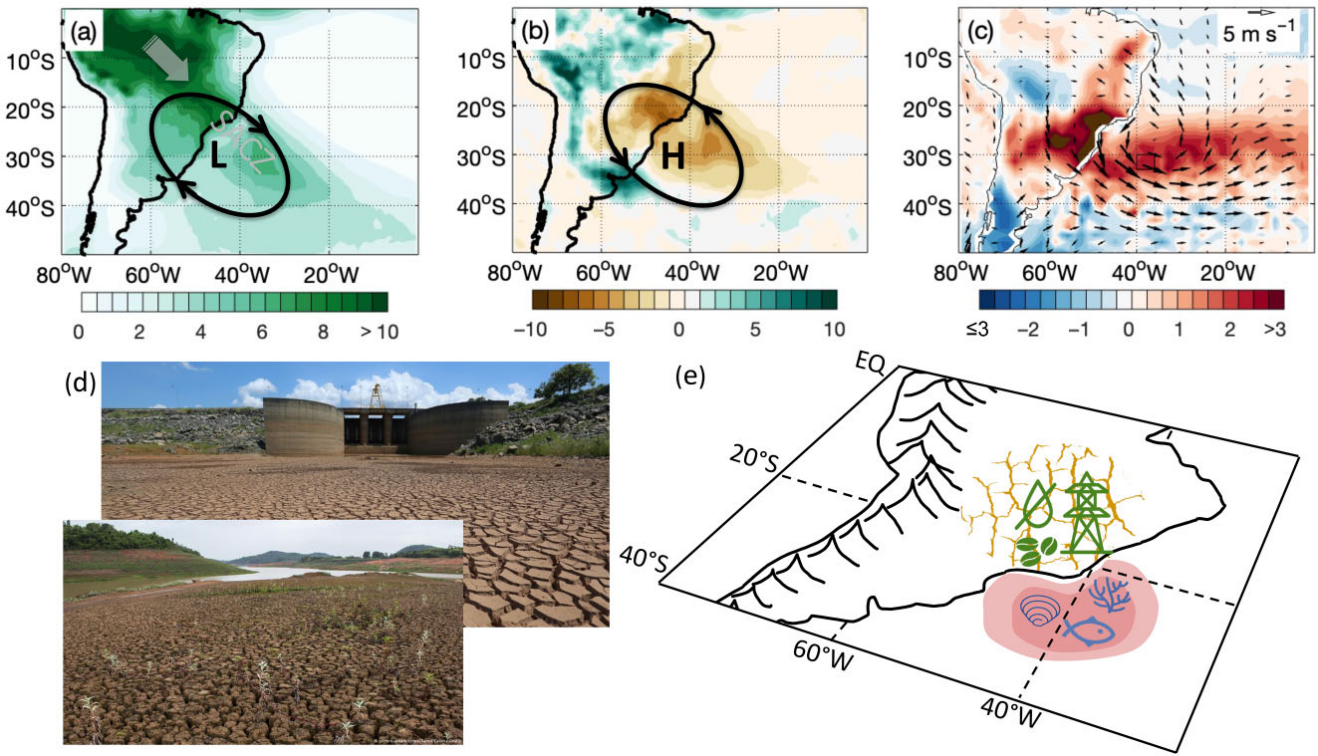
The 2013/14 compound extreme event: (a) precipitation (mm/day) averaged over austral summer (December–March) for the period 1979–2020; (b) anomalies of precipitation averaged over the summer of 2013/14; (c) anomalies of sea surface and air temperature (shading,°C) and winds (vectors, m/s) averaged over the summer of 2013/14; (d) images of the main water supplier for São Paulo city (Cantareira reservoir) and crop failure during the summer of 2013/14; and (e) schematic of the consequences of the drought and heatwaves for the region. In (a), the SACZ is a band of strong precipitation associated with a cyclonic circulation (low-pressure center “L”) over eastern South America that channels moisture from the Amazon toward southeast Brazil (solid white arrow). In (b), a persistent anticyclonic circulation (high-pressure center “H”) established itself over southeast Brazil shutting off the climatological moisture flux and leading to the drought and heatwaves. Details of the data used in this figure can be found in the Data Availability Statement.

In the austral summer of 2013/14, eastern South America experienced one of its worst recorded droughts (21, 22). Associated with the drought, extremes of air temperatures occurred over land and at the same time an unprecedented marine heatwave developed in the western South Atlantic (23). These compound extreme events were associated with the failure of the South American Monsoon System, which is characterized by the development of the South Atlantic Convergence Zone (SACZ) during austral summer (Fig. 2a). During the summer of 2013/14, however, the development of the SACZ and its associated rainfall were inhibited, leading to the drought (Fig.   2b). Reduced cloud cover then allowed more solar radiation to reach the surface, leading to land and marine heatwaves in the region (Fig. 2c). The anomalous atmospheric circulation that led to the compound extremes was remotely caused by tropical convection in the Indian and Pacific oceans (21, 22, 23).

This compound extreme event led to water and power shortages in southeast Brazil, a region that is heavily populated, home to more than 80 million people, and responsible for 60% of the Brazilian gross domestic product (Fig. 2d and e). It reduced Brazilian soy, coffee, and sugarcane production, impacting food supplies globally and increasing worldwide prices (24, 25). It decreased the production of oysters and the catch of some commercially important fish species, while decimating clams along the southern coast of Brazil (26). It affected human health by increasing the risks of heat strokes and vector-borne diseases, causing a dengue fever outbreak that tripled the usual number of fatalities (27). In addition, compound events like this have a disastrous impact on ecosystem degradation and loss of land and marine biodiversity.

Clearly, if this event is in any way a sign of things to come from climate change (see (28)), it would have major implications for climate adaptation in southeast Brazil. However, answering this question is not easy. The proximate cause of the event was the anticyclonic circulation anomaly, which inhibited the seasonal development of the SACZ, inducing both the drought and the land and marine heatwaves. Although such circulation anomalies appear to have become more frequent and intense in recent decades, along with concomitant changes in precipitation (29, 30), the observational record is not long. With regard to guidance from climate models, rainfall variability in this region is not well represented in its main observed characteristics such as high percentiles, seasonality, and spatial variability (31). This may reflect the fact that the models are unable to reproduce the transition between tropical and subtropical climates in this region as well as the passage of frontal systems. Moreover, rainfall variability over eastern South America is strongly affected by remote phenomena ranging from subseasonal to decadal time scales, such as the Madden–Julian Oscillation and El Niño–Southern Oscillation (32, 33), as was the case for this event. Yet climate models largely remain unable to realistically simulate these phenomena and their teleconnections. When it comes to the expected response to climate change, the climate models give a mixed signal over this region in its wet summer season, with some models simulating a robust wetting and some a robust drying (see Fig. 2a of (34)).

This situation, with limited long-term data records, a poor representation in climate models of the relevant physical processes behind extreme events, and ambiguous model predictions of the forced response to climate change, is not specific to this region and is particularly characteristic of countries in the Global South (13, 34). This is illustrated by Figure SPM.3 of IPCC (35), which explicitly identifies the regions for which there is limited data and/or literature on past changes, and/or low confidence in the human contribution to those changes due to limited evidence.

## Grappling with complexity

We start from the point that climate-change science is anchored in physics, which seeks general explanations based on fundamental physical principles such as the first law of thermodynamics. This foundation underpins consensus statements on climate change and is sufficient to justify urgent action on climate mitigation and the imperative to reach net zero greenhouse gas emissions as soon as possible. Such a framing of climate information corresponds to what Stirling (36) characterizes as ‘‘singular, definitive.’’ However, when it comes to information for climate adaptation, which is inherently local, the significant uncertainty in the response of atmospheric circulation to climate change, including the dynamical conditions conducive to weather or climate extremes, precludes confident statements (37). Here, the state of knowledge is more accurately characterized as ‘‘plural, conditional’’ (36), as exemplified by our case study. The complexity is only exacerbated for climate impacts, leading to what has been described as a ‘‘cascade of uncertainty’’ (38). Stirling (36) argues that under such conditions, scientists should resist the pressure (or temptation) to create singular, definitive statements, and “keep it complex”. Our first quotation from Schumacher echoes this call:

“G.N.M. Tyrell [a British mathematician, physicist, radio engineer, and parapsychologist (1879–1952)] has put forward the terms ‘divergent’ and ‘convergent’ to distinguish problems which cannot be solved by logical reasoning from those that can… The physical sciences and mathematics are concerned exclusively with convergent problems… The price, however, is a heavy one. Dealing exclusively with convergent problems does not lead into life but away from it.” (p. 76; page numbers for quotations are from the 2011 Vintage version of Schumacher (39).

We can re-interpret Schumacher's ‘‘convergent’’ problems—not to be confused with the very different NSF concept of Convergence Research (https://www.nsf.gov/od/oia/convergence/index.jsp)—as those amenable to a ‘‘singular, definitive’’ framing of climate information, and his ‘‘divergent’’ problems as those requiring a ‘‘plural, conditional’’ framing. The implication is that if climate scientists are to institutionalize research as care, they need to be prepared to grapple with problems that do not have a clear answer and require a ‘‘plural, conditional’’ framing of climate information, including multiple perspectives (18).

Yet, climate-change science as it is currently practiced tends to frame the scientific questions as ‘‘singular, definitive’’ ones. It does this by focusing on projections from climate models and emphasizing where there is consensus amongst them, acknowledging uncertainty but not really exploring it. Consensus is achieved by a focus on certain kinds of phenomena (e.g. extreme temperatures on either daily or seasonal timescales, rather than on multiweek timescales, which may be more impactful but are less reliably represented in climate models) or through spatial aggregation. Shepherd (40) characterizes this approach as a focus on reliability over informativeness. The use of ‘‘risk indices’’ based on meteorological fields alone, as a proxy for climate impacts, is another common way to achieve a ‘‘singular, definitive’’ framing, yet it is well-recognized that extreme meteorology does not necessarily correspond to extreme impact (41). Finally, homogenized and gridded data products, whilst they facilitate scientific analysis and certainly have their role, can lead to artefacts from temporal inhomogeneities, such as spurious trends (42). This is indeed an issue for precipitation trends in the region of our case study. In that case, local station data may be more informative, although would not be considered to be of the same scientific quality as the homogenized and gridded products, and might be difficult to include in scientific publications.

The focus on ‘‘singular, definitive’’ problems is also expressed in the still common practice across most published climate-change science to interpret ‘‘statistical significance’’ in a dichotomous true/false manner, and avoid the articulation of multiple plausible hypotheses, which would allow a ‘‘plural, conditional’’ framing of uncertainty (43). This is particularly apparent in the practice of probabilistic event attribution, which is the most common form of event attribution. In a probabilistic event attribution of the drought described in our case study (albeit analyzing the longer period 2012–2016), Martins et al. (44) found “insufficient evidence” that climate change increased drought risk. We might well ask, “insufficient for whom?”. What they meant was insufficient to reject the null hypothesis of no effect from climate change. This conclusion was based on the fact that two of the climate models used (which were from the same model family) predicted a decrease in drought risk, and that when combining estimates from observations and models, the uncertainty straddled zero anthropogenic effect (see their Fig. 13.2(i)).

However, by adopting such a ‘‘singular, definitive’’ framing, this reasoning not only violates several logical errors (43), but also avoids the important question, “insufficient for whom?” In fact, the observed record suggests increasing drought risk in this region (see Fig. SPM.3c of (35)), and both the precipitation trends shown in Fig. 13.2(i) of Martins et al. (44), as well as the CMIP5 model results reported there, point toward increasing precipitation deficit. Given the observed trends, and the climate models’ inability to adequately represent rainfall variability in this region, can we afford to wait until we have more confident attribution statements to provide decision-makers with the information necessary to plan and adapt? The focus on statistical significance rather than relevance for decision-making is an example of what Schumacher describes as “[deriving] ‘‘reality’’ from a conceptual framework, instead of deriving a conceptual framework from reality” (p. 240).

How, then, to represent relevant knowledge in the face of such uncertainty? As Schumacher says, “The future cannot be forecast, but it can be explored” (p. 201). In particular:

“We can [talk with certainty] about future events only on the basis of assumptions. In other words, we can formulate conditional statements about the future… [Such a statement is] not a forecast or prediction…but an exploratory calculation, which, being conditional, has the virtue of mathematical certainty.” (p. 190)

Since climate-change science in anchored in physics rather than mathematics, we can replace Schumacher's ‘‘mathematical certainty’’ with ‘‘singular, definitive’’ knowledge. Each conditional statement can be ‘‘singular, definitive’’, but together the representation of knowledge is ‘‘plural, conditional’’. That future climate and the space for future action are conditional on anthropogenic climate forcings and socio-economic structures, respectively, is not in any way controversial for climate scientists, and is represented in the RCP–SSP scenarios (45) used by the IPCC for its analysis. However, the standard approach of both physicists and economists is to make general predictions (known as projections) that are conditional on those scenarios. Schumacher is challenging us to go further and to represent *all* our knowledge in conditional form. Such an approach grapples with complexity by accepting that nature is governed by a patchwork of laws (14), knitting together different kinds of knowledge within a conditional framework. There exists a growing set of tools for doing so, within a framework known as Decision Making under Deep Uncertainty (46).

## The importance of simplicity

Climate-change science is, without a doubt, big science. The satellite-based measurement systems which provide a global perspective on the changing planet are massive scientific and technical efforts, while the climate simulation models which embody our knowledge of physical laws represent human and technological investments on a similar scale. No climate-change scientist would seriously suggest that we could do without these assets. But that does not mean they are sufficient. It is widely recognized that satellite-based measurements are generally quite indirect inferences, heavily dependent on mathematical models of the measurement process, and need to be complemented by in situ measurements. When it comes to climate models, their unquantifiable uncertainties mean that whilst the models need to be taken seriously, they should not be taken literally (47). Despite the big science behind measurements and models, we are left with a patchwork of knowledge.

Railing against big technology (rather than big science), Schumacher wrote:

“Today, we suffer from an almost universal idolatry of giantism. It is therefore necessary to insist on the virtue of smallness—where this applies.” (p. 49)

Here, we have the essence of *Small is Beautiful*. Yet the last three words in the quotation are crucial: big approaches have their place; it is just that there needs to be an appropriate balance between big and small approaches, and the balance will depend on the decision-making context. Schumacher argued that economics was out of balance in this respect; we argue the same today for climate-change science for adaptation. How can such an imbalance arise? Schumacher offers several clues:

“It is a fixation in the mind, that unless you have the latest [technology] you can't do anything at all, and this is the thing that has to be overcome.” (p. 182) “…it is rather more difficult to recapture directness and simplicity than to advance in the direction of ever more sophistication and complexity” (p. 127) “…in modern times all too little attention has been paid to the study of ideas which form the very instruments by which thought and observation proceed” (p. 63).

All these statements ring true in the context of climate-change science, and point to the importance of simplicity. This might seem like a contradiction with the call of the previous section to grapple with complexity, but it is not. The point is that in order to respect the complexity of the real world, our methods of analysis need to remain simple enough to be interpretable and open to interrogation, rather than offering what Parker and Risbey (48) describe as ‘‘false precision’’ (see also (4)). As Schumacher notes:

“Crude methods of forecasting…are not likely to lead into the errors of spurious versimilitude and spurious detailing” (p. 195) “If the forecasts were presented quite artlessly, as it were, on the back of an envelope, [the person using the forecasts] would have a much better chance of appreciating their tenuous character and the fact that, forecasts or no forecasts, someone has to take an entrepreneurial decision about an unknown future.” (p. 196)

**Fig. 3.**
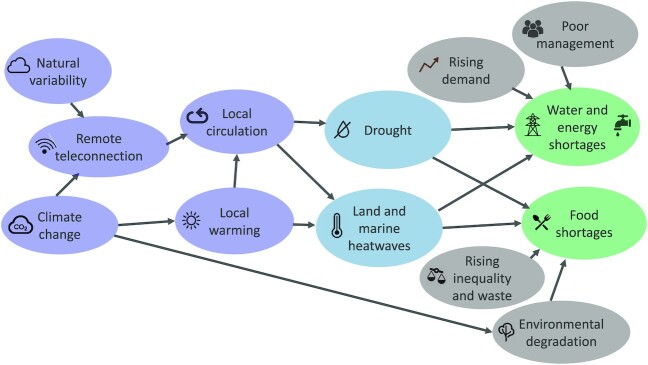
Causal network for the case study illustrated in Fig. 2. Arrows indicate direction of causal influence but can include the effects of feedbacks. The purple shading indicates elements whose causality lies in the weather and climate domain, the blue shading indicates the hazards, the gray shading exposure and vulnerability, and the green shading the impacts. Causal networks allow one to navigate the cascade of uncertainty involved in such a complex risk landscape through the device of conditionality, which means specifying the state of some of the nodes in the network. By, thereby, seeking conditional rather than unconditional predictions, counter-factual outcomes and system sensitivities can be explored in a targeted way. See text for further details.

The relevance of this statement in our context is obvious. In the previous section, we argued for representing relevant knowledge about climate adaptation and local climate risk in a conditional manner. Shepherd (40) calls this the ‘‘storyline” approach to regional climate information, and suggests the use of causal networks since they are grounded in Bayesian (conditional) logic (49). In contrast to data-driven methods such as machine learning, which are challenged by physical consistency and hence by physical interpretability (50), causal networks incorporate domain knowledge and can be used to explore counter-factual situations in a straightforward manner (51). Fig. 3 represents our case study from this perspective. The devastating impacts, which spanned the water–energy–food security nexus, were caused by both drought and heatwaves, which themselves were the result of local circulation conditions, mediated by cloud cover and rainfall. This introduces a strong element of correlated risk, which storylines are specifically designed to represent. For example, a physical climate storyline with local meteorological fidelity could be constructed to represent the possibility of a weakening SACZ and decreasing summertime precipitation in this region, to explore high-impact outcomes and avoid the sort of false-negative errors that the probabilistic event attribution described in the previous section is prone to (40, 52). But at the same time, the role of nonclimatic factors such as those indicated in gray shading in Fig. 3 could also be considered, and potentially even modeled if suitable modeling tools were available. In this way, both local liability and the value of future adaptation options for risk reduction can be considered. A strong benefit of the storyline approach (and of Bayesian reasoning more generally) is that causality is not represented in a dichotomous manner. Rather, the role of multiple causal factors can be explicitly quantified in a logically self-consistent manner.

Causal networks are an expression of conditional hypotheses (53). Their structure is, thereby, explicitly subjective (i.e. dependent on which hypotheses are imposed, which is a matter of expert judgement, and thus reflects the particular perspective of the knower), which goes against the desire by climate scientists to be objective. So-called ‘‘frequentist’’ methods of analysis may give the appearance of objectivity, but they only achieve this by burying subjective choices and by adopting rules of inference which have no basis in logic and can violate common sense (43). In the end, there is no avoiding bringing values into climate-change science, and no such thing as ‘‘pure’’ objectivity (54). The goal is, rather, transparency and logical rigor. This argues strongly for Bayesian approaches, the results of which are much easier for decision-makers to interpret (55). How far one chooses to go down the Bayesian road will depend on the context, but at the very least, one needs to clearly specify one's hypotheses and the evidence to be considered (or not considered), and anchor the analysis in physical knowledge. That makes risk assessment more than a tick-box exercise, which aligns with Schumacher's emphasis on the importance of ideas over sophisticated technologies (see earlier quotations).

## Empowering local communities

The impacts of climate change are global in scope, but are ultimately felt at a local scale. It is, therefore, essential that local communities have the means to make sense of their own situation, and use that understanding to inform local decision-making. However, their ability to do so depends on what is deemed to constitute reliable knowledge. It follows that this issue is inseparable from the themes of the two previous sections. The IPCC detection-attribution framework (56) explicitly states that scientific analysis of climate change should not be motivated by observations, but instead by process understanding (which is generally interpreted as model projections); and that confounding factors need to be adequately controlled for. Both recommendations are entirely orthodox from a scientific perspective, but the inevitable result is to disenfranchize local, contextual knowledge. If the only form of reliable knowledge is considered to be general (rather than conditional) and produced by sophisticated tools, then there will inevitably be a power imbalance between rich and poor. This is most clearly apparent between Global North and Global South (57), but it will also be the case within-country, and even between different communities occupying the same region but with different levels of vulnerability.

Exactly the same issue arises in economic development, of course, and was of great concern to Schumacher. His first appeal was to meaning-making (for which he used the word ‘‘intelligible’’):

“When a thing is intelligible you have a sense of participation; when a thing is unintelligible you have a sense of estrangement.” (p. 65)

This statement recognizes that the first step to empowerment is a sense of participation, and therefore, of agency. And this requires intelligibility, by which we mean the ability to make sense of one's own situation. Intelligibility (and meaning) is achieved partly by respecting the complexity of a local risk landscape, which values local knowledge and devalues generalized or aggregate descriptions; and partly by employing simple methods of representing knowledge. The latter corresponds to what Schumacher calls ‘‘intermediate technology’’, by which he means methods that are simple but not elementary:

“The idea of intermediate technology does not imply simply a ‘‘going back’’ in history to methods now outdated… The real achievement lies in the accumulation of precise knowledge, and this knowledge can be applied in a great variety of ways, of which the current application in a modern industry [namely using the most sophisticated tools available] is only one.” (p. 155)

By ‘‘technology’’ Schumacher was referring to physical equipment; in our case we need to re-interpret ‘‘technology’’ as methods of producing and analyzing climate information. An example of a simple but not elementary analysis method in climate science is the use of causal networks to understand atmospheric teleconnections, which underpin climate variability. In their simplest form causal networks reduce to humble regression, yet they bring together physical and statistical understandings of teleconnections within an intelligible framework (58). They can, therefore, provide a key component of physical climate storylines, to connect global to local aspects of climate change. For example, in our case study, both climate change and natural variability can affect the teleconnections from the Indian and Pacific oceans that lead to local persistent anticyclonic circulation and drought in eastern South America (Fig. 3).

The use of simple analysis methods such as causal networks and storylines not only lets ideas flourish (to return to an earlier point of Schumacher's), and thereby creates intelligibility (or meaning-making), but also democratizes the production of climate information, which is also an important principle for economic development. In his context, Schumacher put it thus:

“It is therefore more important that everybody should produce something than that a few people should each produce a great deal.” (p. 144).

The implication for us is that climate information should be produced by many people, spread around the world, rather than by a small number of experts. In order to achieve this end in the case of technology, Schumacher proposed the following modus operandi:

“The real task may be formulated in four propositions:

- First, that workplaces have to be created in the areas where people live…

- Second, that these workplaces must be, on average, cheap…

- Third, that the production methods employed must be relatively simple…

- Fourth, the production should be mainly from local materials and mainly for local use.” (p. 145)

In our case we are talking about climate information, so local ‘‘workplaces’’ can draw on the entire worldwide storehouse of climate information. What would Schumacher's ‘‘four propositions’’ mean for climate-change science for adaptation? Simply put, intelligible climate-change science for local adaptation must be created locally by the local scientific community with the engagement of a community of users from the beginning of the production process (7), using accessible and simple tools. Not only does this ensure intelligibility, but most importantly it takes into consideration forms of expert knowledge based on operational and local experience (59). An example is the Household Economy Approach (60), which uses detailed socio-economic data to simulate the effect of climate and other shocks on household income and food access, and is widely used by governments in Africa for decision-making to avoid impoverishment and erosion of household resilience. Young et al. (61) show how this approach can be embedded within a causal network to develop storylines of climate-related food security risk for a particular region of Namibia.

Deriving the conceptual framework from reality, rather than the other way around, finesses the oft-mentioned problem of “bridging the gap between complex risk information and decision-making” or “translating complex risk information for decision-makers.” In particular, climate information is brought directly into the decision-making framework. This is particularly important for the Global South, where human resources, funding, and systematic data are limited, whilst local knowledge is rich and abundant. Yet the same situation arises in regions within the Global North as well. For example, in the Canadian Arctic, the standard data products for environmental conditions can be difficult to use in the field or to combine with more trusted indigenous knowledge (62). Incorporation of indigenous knowledge from the outset in co-development of data products can provide more useable climate information, thereby empowering local communities to make sense of their situation and argue their case in contested decision-making contexts (63).

Challenges with regard to equity and legitimacy should be addressed by creating frameworks that enable the establishment of equitable partnerships. This requires a shift in perspectives on, and processes related to, the design, implementation, and evaluation of success (64). We propose a structure consisting of a nonhierarchical ecosystem of communities of practice—like a mycorrhizal network, sharing resources rather than competing for them, and anchored by ‘‘mother trees’’ (65). In this way, long-term, equitable, and trusting partnerships can be nurtured through a variety of mechanisms, including training, thereby empowering local communities to produce their own climate information and acquire a sense of intelligibility and participation. For this to happen, however, continuity is crucial. The rapid turnover in many organizations (e.g. government ministries) works against the use of ideas and places an emphasis on numbers, which are easily transferred from person to person. This also devalues local, contextual knowledge, and thus must be resisted.

## Conclusion

We have made great progress in climate science, and today we can comfortably say that climate change is real and is unequivocally driven by human activity. Climate science also tells us that we should limit human-induced global warming to a specific level, and this requires strong reductions in greenhouse gas emissions. Mitigation to achieve our vital goals, we know, requires in turn global coordination and government policies. Thus ‘‘top-down’’ approaches to climate information serve us well for mitigation goals.

However, there is a widely accepted gap between the production and use of climate information for adaptation. It is also widely accepted that at least part of the reason for this situation lies in the challenge of bridging between ‘‘top-down’’ approaches to climate information on the global scale, and local decision contexts which necessarily take a ‘‘bottom-up’’ perspective, in which climate change is just one factor among many to consider. We urgently need climate-change science to guide adaptation policies that will minimize the vulnerability of societies across the world by reducing exposure and sensitivity to climate hazards and by enhancing the capacity of communities to proactively adapt to evolving climate risks. We also know that many of the adaptation policies have mitigation benefits as well.

We need to grapple with the complexity of local situations, our local reality. However, simplicity is important when dealing with deep uncertainty. We need to empower local communities to be able to make sense of their own situation, which can be addressed by developing methodologies for producing and analyzing climate information that build trust and transparency. As Schumacher says:

“Needless to say, wealth, education, research, and many other things are needed for any civilization, but what is most needed today is a revision of the ends which these means are meant to serve.” (p. 249)

Much of climate-change science is necessarily big science. But in order to make climate information useable for adaptation, it is also necessary to discover the beauty of smallness. Again, Schumacher enlightens us:

“Science and engineering produce know-how; but know-how is nothing by itself; it is a means without an end, a mere potentiality, an unfinished sentence. Can education help us to finish the sentence, to turn the potentiality into a reality to the benefit of man?” (p. 62)

To translate this into our context: science produces climate information; but climate information is nothing by itself; it is a means without an end, a mere potentiality, an unfinished sentence. Can this new paradigm, of *climate-change science as if people mattered*, help us to finish the sentence, to turn the potentiality into reality to the benefit of all?

## Funding

T.G.S. acknowledges funding from the European Union's Horizon 2020 Research and Innovation Programme under the grant agreement number 820712 (RECEIPT). R.R.R. is supported by Rede CLIMA (FINEP 01.13.0353-00) and INCT-MCII (FAPESP 2014/50848-9, CAPES 88887.136402-00, and CNPq 465501/2014-1).

## Data Availability

The SST and atmospheric data used in Fig. 2 are freely available from www.esrl.noaa.gov/psd/data/gridded/data.noaa.oisst.v2.highres.html (66) and http://apps.ecmwf.int/datasets/data/interim-full-daily/levtype=sfc/(67), respectively. The precipitation data are provided freely at www.esrl.noaa.gov/psd/data/gridded/ (68).
